# The PAMPA datasets: a metagenomic survey of microbial communities in Argentinean pampean soils

**DOI:** 10.1186/2049-2618-1-21

**Published:** 2013-07-29

**Authors:** Nicolás Rascovan, Belén Carbonetto, Santiago Revale, Marina D Reinert, Roberto Alvarez, Alicia M Godeas, Roxana Colombo, Mario Aguilar, María Victoria Novas, Leopoldo Iannone, Alicia M Zelada, Alejandro Pardo, Gustavo Schrauf, Alejandro Mentaberry, Martín P Vazquez

**Affiliations:** 1 Instituto de Agrobiotecnología de Rosario (INDEAR), Ocampo 210 bis, Predio CCT Rosario, Santa Fe 2000, Argentina; 2 Facultad de Agronomía, Universidad de Buenos Aires, Av. San Martin 4453, Buenos Aires 1417, Argentina; 3 Departamento de Biodiversidad y Biología Experimental, Facultad de Ciencias Exactas y Naturales, Universidad de Buenos Aires, Ciudad Universitaria, 4to Piso, Pabellón 2, Buenos Aires 1428, Argentina; 4 Instituto de Biotecnología y Biología Molecular (IBBM), Universidad Nacional de La Plata-16 CONICET, La Plata, Argentina; 5 PROPLAME-PRHIDEB-CONICET - Departamento de Biodiversidad y Biología Experimental, Facultad de Ciencias Exactas y Naturales, Universidad de Buenos Aires, Ciudad Universitaria, 4to Piso, Pabellón 2, Buenos Aires 1428, Argentina; 6 Laboratorio de Agrobiotecnología, Departamento de Fisiología y Biología Molecular y Celular, Facultad de Ciencias Exactas y Naturales, Universidad de Buenos Aires, and Consejo Nacional de Investigaciones Cientificas y Técnicas (CONICET) Intendente Güiraldes 2160, Ciudad Universitaria, Buenos Aires 1428, Argentina; 7 Laboratorio de Micología Molecular, Departmento de Ciencia y Tecnología, Universidad Nacional de Quilmes and Consejo Nacional de Investigaciones Cientificas y Técnicas (CONICET), Roque Saenz Peña 352 Bernal, Buenos Aires B1876BXD, Argentina

**Keywords:** Soil microbial communities, Shotgun metagenome sequencing, Amplicon sequencing, Argentina, Pampas, Land use

## Abstract

**Background:**

Soil is among the most diverse and complex environments in the world. Soil microorganisms play an essential role in biogeochemical cycles and affect plant growth and crop production. However, our knowledge of the relationship between species-assemblies and soil ecosystem processes is still very limited. The aim of this study was to generate a comprehensive metagenomic survey to evaluate the effect of high-input agricultural practices on soil microbial communities.

**Results:**

We collected soil samples from three different areas in the Argentinean Pampean region under three different types of land uses and two soil sources (bulk and rhizospheric). We extracted total DNA from all samples and also synthetized cDNA from rhizospheric samples. Using 454-FLX technology, we generated 112 16S ribosomal DNA and 14 16S ribosomal RNA amplicon libraries totaling 1.3 M reads and 36 shotgun metagenome libraries totaling 17.8 million reads (7.7 GB). Our preliminary results suggested that water availability could be the primary driver that defined microbial assemblages over land use and soil source. However, when water was not a limiting resource (annual precipitation >800 mm) land use was a primary driver.

**Conclusion:**

This was the first metagenomic study of soil conducted in Argentina and our datasets are among the few large soil datasets publicly available. The detailed analysis of these data will provide a step forward in our understanding of how soil microbiomes respond to high-input agricultural systems, and they will serve as a useful comparison with other soil metagenomic studies worldwide.

## Background

The Argentine Pampas is a plain area of 60 million ha. Because of its large expanse and high yields, it is one of the most productive areas for grain crop production in the world [1]. Indeed, 90% of the pampean surface is currently used for high-input agricultural purposes. Argentina is currently the third and fourth world producer of soybean and maize, respectively [2]. This production is mostly concentrated in the pampean region.

Since 1980, agriculture has rapidly expanded in the region, replacing grasslands, with the widespread adoption of limited tillage systems, particularly no-till with crop rotation [3]. These practices have been reported to preserve surface water, prevent soil erosion and return nutrients to soil [4-6]. However, concerns remain regarding the impact of these practices on soil quality, microbial diversity and community assemblages.

Changes in microbial communities throughout the Argentine Pampas are poorly reported. Most studies have focused on the tillage effects on microbial biomass or specific microbial activities such as the utilization of specific substrates, extracellular enzyme production, or mineralization [7-9]. Other studies have focused on well-studied and particular bacterial taxa rather than the microbial community structure itself [10,11]. Studies conducted with an ecological approach have usually focused on the individual effects of land use such as the application of herbicides [12,13]. In such cases, community variability was assessed using classical fingerprinting techniques (such as RFLP and DGGE), which only capture the most dominant species in the environment [14,15]. In this regard, classical approaches are inadequate for describing highly diverse soil microbial communities.

High-throughput sequencing (HTS) has opened a new era for environmental microbial studies as large amounts of genetic information can be obtained without culturing. Some recent studies have used amplicon and shotgun metagenome pyrosequencing to characterize soil microbial communities worldwide [16-20]. These strategies have allowed a more exhaustive characterization of community patterns, composition and metabolic capabilities, and continue to change our understanding of the microbial world. To date, however, HTS approaches have not been employed in Argentina as a means to compare tillage systems and evaluate land use effects on soil microbial communities.

**Figure 1:**
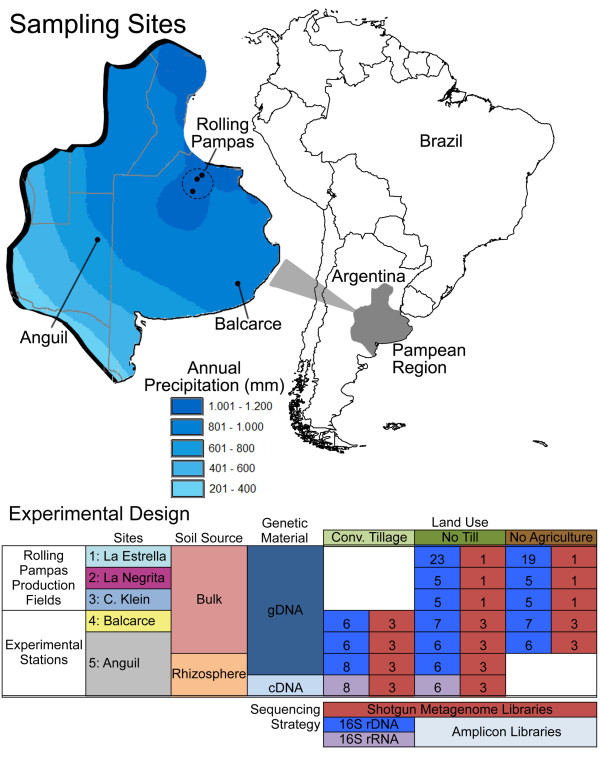
**Sampling sites and experimental design for PAMPA datasets.** The geographic location of the Argentinean Pampas is marked in grey on the map of South America. The isohyets in the region are shown in blue (top left). Soil samples were taken in three different isohyets and are indicated with numbers (1: La Estrella, 2: La Negrita, 3: Criadero Klein in the wet rolling pampas region, 4: Balcarce, a semi-wet region, 5: Anguil, a semi-arid region). The experimental design is indicated in a table below the map. Soil source, genetic material, land use and sequencing method are indicated for each sampling site. The number of replicates per sample analyzed by each sequencing method is shown inside the boxes. Additional and detailed information on each type of library per sampling site can be found in Additional file 2: Table S1. gDNA, genomic DNA; rDNA, ribosomal DNA; rRNA, ribosomal RNA.

In this study, we examined the impact of agricultural management on soil microbial communities. To do so, we collected soil samples from sites under three different types of land use (conventional tillage, no till and no agriculture), at each of five different locations in the Argentine Pampas region (Figure 1). From these samples, we generated amplicon and shotgun metagenome libraries, which were subsequently sequenced using 454-FLX pyrosequencing. Together these data compose the designated PAMPA datasets.

## Methods

Soil samples were obtained at five different sites in the Argentinean Pampas located in three isohyet regions (Figure 1): three production fields in the rolling pampas (La Estrella: LE, La Negrita: LN, Criadero Klein: CK, wet weather, 1,000 to 1,200 annual mm) and two experimental stations, at Balcarce (Ba, semi-wet, 800 to 1,000 annual mm) and Anguil (An, semi-arid, 600 to 800 annual mm). At each experimental station, soils were collected from three plots, with three different types of land use: conventional tillage (CT), no till (NT) and soils with no agricultural (NA) management. Bulk soil was obtained from all plots included in this study. In addition, wheat rhizospheric soil was also obtained from the Anguil CT and NT plots. Only one sampling campaign was performed at each site, except at the La Estrella production field in the rolling pampas where there were six sampling time points over a year. At least two independent soil samples from each plot and land use site were collected, resulting in a total of 30 samples for Anguil station, 20 for Balcarce station and 62 for the rolling pampas region (see Additional files 1 and 2 for a detailed description of sampling strategy and sample processing). Total DNA was prepared from all soil samples. In addition, total cDNA was also prepared from Anguil rhizospheric samples. Amplicon sequencing libraries were constructed by PCR amplification of the V4 variable region in the 16S rRNA gene. Shotgun metagenome libraries were also constructed from one genomic DNA (gDNA) (and one cDNA, when available) sample obtained from each plot (see Additional files 1 and 2 for further details). Amplicon and shotgun libraries were sequenced using 454-FLX-Titanium chemistry. Raw data processing was performed following standard procedures suggested by the manufacturer.

We obtained a total of 19,325,913 reads and 7,740,811,541 bases from 30 samples by 454-FLX shotgun metagenome sequencing and 1,051,470 16S ribosomal DNA and ribosomal RNA (rDNA/rRNA) reads from 126 samples by amplicon sequencing. The metatranscriptomic shotgun libraries were excluded from the analysis due to the low number of reads recovered after rRNA trimming (more than ten fold below other samples). The amplicon dataset was analyzed using QIIME v1.5 software package [21]. Shotgun metagenome datasets were annotated by BLAST against the NCBI database and subsequent results imported into MEGAN [22] for further analysis. Numerical and statistical analyses were performed using the METAGENassist software [23] and the R packages ‘BiodiversityR’ and ‘Vegan’ (R Development Core Team) (see Additional file 1).

## Quality assurance

To rule out possible contaminants from non-microbial species, such as plant, human or any other allochthonous DNA, in our metagenome shotgun libraries, a taxonomy assignment of all reads was assessed. We performed peptide prediction using FragGeneScan [24] followed by BlastP annotation against the NCBI Database. The Blast output was analyzed using MEGAN [22]. The results showed that 95% of the classified sequences were identified as Bacteria, 1% as Eukarya and 0.6% as Archaea, whereas the remaining 3.4% of sequences could not be classified above the cellular organism level (data not shown). Within the Eukarya, 42% of reads were classified as Viridiplantae (plants), 27% as Fungi, 12% as Metazoa, 6% as diatoms and 13% to other groups or could not be classified (data not shown). Plant sequences are likely to be from decomposing material. These results suggest that contamination with allochthonous DNA is minimal or nonexistent as we could not identify any genetic material from unexpected species in the soils (for example, humans).

## Initial findings

**Figure 2:**
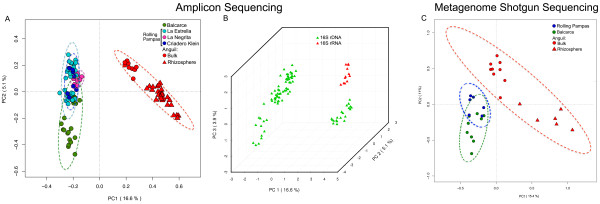
**Principal component analysis. (A)** A total of 103 soil samples were analyzed by 16S rDNA/rRNA V4 amplicon sequencing. Sequences were clustered in OTUs at 90% similarity. Low abundance and infrequent OTUs were excluded from the analysis (see Additional file 1 for a detailed description of the filtering procedures). Datasets were normalized before PCA. **(B)** Differences among 16S rDNA and rRNA were evident in the first three axes of the PCA analysis. **(C)** A total of 30 soil samples were analyzed by metagenomic shotgun sequencing. Predicted peptides were annotated by BlastP against the NCBI database and the results assigned to categories in SEED Database. Low abundance and infrequent SEED categories were excluded from the analysis (see Additional file 1). Datasets were normalized before PCA. OTU, operational taxonomic unit; PCA, principal component analysis; rDNA, ribosomal DNA; rRNA, ribosomal RNA.

We found that geographic-specific differences, possibly associated with water availability, were evident in the 16S rRNA amplicon analysis of 103 soil communities (23 samples were excluded from the preliminary analysis due to differences in sequencing depth and other biases, see Additional files 1 and 2). The semi-arid soils (An) harbored communities that clustered separately from the wet (LE, LN, CK) and semi-wet (Ba) soil microbial communities (analysis of similarity: ANOSIM = 0.672, *P* < 0.001, Figure 2A, Additional file 3: Figure S1). This observation could be explained by the very different environmental conditions in both areas: the eastern area (wet and semi-wet) is humid and fertile with fine-textured soils that are rich in organic matter, while the western area is semi-arid with shallow coarse-textured soils with low levels of organic matter. We used Bioenv analysis (see Additional file 1 for further details of the analysis) to test which soil properties best explained the variation in microbial community structure. We found that clay, organic matter content, pH and salinity were the most influential variables (Mantel test: *r* = 0.6209, *P* = 0.001).

Differences in microbial communities within the semi-arid region (An) were largely determined by soil source, that is rhizospheric compared to bulk soil (ANOSIM = 0.5614, *P* < 0.001, Figure 2A, Additional file 3: Figure S1). In addition, rhizospheric samples clustered separately depending on the type of genetic material amplified (ANOSIM = 0.5169, *P* = 0.001, Figure 2B, Additional file 3: Figure S1). At the DNA level, active, inactive and even dead microorganisms were detected, that is, all the microbes present in the sample. However, at the RNA level, only metabolically active microorganisms were detected due to their high rates of rRNA expression. Our results show that rhizospheric microbial signatures detected by 16S rDNA are clearly distinct from those detected by 16S rRNA, suggesting that bacterial activity was not necessarily correlated with bacterial abundance.

Land use was another important driver that defined microbial community assemblages. Bulk soil samples clustered separately depending on land use (ANOSIM: Anguil = 0.3954, *P* = 0.017; Balcarce = 0.3795, *P* = 0.001; rolling pampas = 0.2072, *P* = 0.01, Additional file 3: Figure S1). Moreover, samples collected from soils under different tillage systems at the two experimental stations (Ba, An) also clustered separately in the analysis (ANOSIM: Balcarce = 0.5476, *P* = 0.001; Anguil = 0.2652, *P* = 0.001, Additional file 3: Figure S1). These results suggest that different microbial communities were selected under each type of soil management.

The evaluation of metabolic categories using the shotgun metagenome libraries also showed that semi-arid western locations were different from wet and semi-wet eastern sites (ANOSIM = 0.2806, *P* < 0.001). Therefore, we propose that water availability is probably the primary driver that shapes microbial communities (Figure 2C, Additional file 3: Figure S1). There was also clear separation by soil source in western semi-arid samples (ANOSIM = 0.6688, *P* < 0.001, Figure 2C, Additional file 3: Figure S1). In addition, bulk soil samples clustered separately according to tillage system in An and Ba (ANOSIM: Balcarce = 0.5391, *P* = 0.01; Anguil = 0.2346, *P* = 0.02, Additional file 3: Figure S1). However, the latter observation was less defined for rhizospheric samples, suggesting that other conditions, such as plant phenotype and exudates, could determine bacterial populations in rhizospheric communities. The soil properties that best explained the functional variation between samples for shotgun sequencing analysis were silt, organic matter, nitrogen content, pH and salinity (Mantel test: *r* = 0.2771, *P* = 0.002).

Even though additional work is required, preliminary results indicated that differences in microbial communities were largely defined by the variables considered, for example, water availability, geographic location, soil source, genetic material amplified and land use or tillage system. However, this was not always observed at the functional metagenomic level, since some samples showed patterns different from those in amplicon analysis (Additional file 3: Figure S1). Differences between the amplicon and shotgun analyses could be due to the fact that the 16S rDNA/rRNA operational taxonomic unit (OTU) analysis was performed by clustering sequences based on similarity, while the metagenomic analysis was based on sequence annotation, constrained by SEED database size, its limited number of categories and their ambiguity in sequence identity. Nevertheless, we could not rule out the possibility that very different microbial species could have similar metabolisms, thus minimizing the differences at metabolic level.

## Future directions

The present project represents the first large-scale metagenomic study of soils in Argentina that explores the link between agricultural management and soil microbiome. The resulting PAMPA datasets are among the few available soil metagenomic datasets based on high-throughput sequencing [17] and, here we presented a preliminary analysis of our data. While more detailed analysis will be needed to test the ideas presented in this paper, results so far have shed considerable light on the largely unknown soil micro-ecosystem of the Argentine Pampas. We showed that the soil microbiome changes primarily because of water availability and agricultural land use, and that these changes are also linked to different tillage systems (no-till or conventional tillage).

Additional analysis of the PAMPA datasets will continue to expand our knowledge of soil microbiome composition and function. Future efforts will be directed at identifying particular species and metabolisms associated with each tillage system in each geographic region and enriched by the rhizosphere. In addition, the PAMPA datasets can also be used in future worldwide soil metagenomic projects for comparative purposes. Additional experimental and sequencing efforts will be needed to describe in detail the root-associated microorganisms for different crops in different conditions. Understanding soil microbial dynamics and identifying specific plant-interacting microbes will be important steps towards improving current agricultural and soil sustainability practices.

## Availability of supporting data

All data are publicly available and can be accessed through the Bioproject PRJNA178180 or directly by the NCBI Sequence Read Archive (SRA) under the accession numbers SRA058523 and SRA056866 (Additional file 2: Table S1 for detailed information). Additional information to that presented in this paper will be available from the Soil Genetic Network (SoilGeNe) website [25].

## Abbreviations

An: Anguil; ANOSIM: analysis of similarity; Ba: Balcarce; CK: Criadero Klein; CT: conventional tillage; gDNA: genomic DNA; HTS: high-throughput sequencing; LE: La Estrella; LN: La Negrita; NA: no agriculture; NT: no till farming; OTU: operational taxonomic unit; PCA: principal component analysis; PCR: polymerase chain reaction; rDNA: ribosomal DNA; rRNA: ribosomal RNA.

## Competing interests

The authors declare that they have no competing interests.

## Authors’ contributions

NR, BC and RA did the sampling. NR, BC and MR processed the samples in the lab and did the sequencing using the 454-FLX. SR performed the bioinformatic processing of sequence data. NR did the analysis, generated preliminary results and uploaded the sequences to the Sequence Read Archive (SRA). BC did some of the statistical analysis. MPV supervised all the work done. NR, BC and MPV participated in the writing of this manuscript. AM supervised all groups involved in this project. All authors participated in the experimental design, discussions about data interpretation and manuscript supervision. All authors read and approved the final manuscript.

## Supplementary Material

Additional file 1 **Supplemental methods.** Detailed description of all materials and methods used to generate and analyze the PAMPA datasets. Click here for file

Additional file 2: Table S1 Metadata for all samples analyzed in the PAMPA datasets. There is a full list of amplicon and shotgun metagenome libraries. Soil types, source of genetic material, sequencing strategies, primers and barcodes used, number of sequences obtained, physicochemical properties and general metadata for each sample are described in detail. Click here for file

Additional file 3: Figure S1 Heatmap and beta-diversity analysis for amplicon and metagenome shotgun libraries in PAMPA datasets. (A) A total of 103 soil samples were analyzed by 16S rDNA/rRNA V4 amplicon sequencing. Sequences were clustered in OTUs at 90% similarity. Low abundance and infrequent OTUs were excluded from the analysis (see Additional file 1 for a detailed description of the filtering procedures). Datasets were normalized and compared using the Pearson distance and Ward clustering algorithm. The scale bar at the top is expressed according to the range of values after normalization. Metadata for each sample are indicated by color bars at the right and references are indicated at the top. (B) A total of 30 soil samples were analyzed by metagenomic shotgun sequencing. Predicted peptides were annotated by BlastP against the NCBI database and the results assigned to SEED categories. Low abundance and infrequent SEED categories were excluded from the analysis (see Additional file 1). Datasets were normalized and compared using the Pearson distance and Ward clustering algorithm. Metadata are indicated with same references as in A. An, Anguil; B, bulk soil; Ba, Balcarce; CK, Criadero Klein; CT, conventional tillage; LE, La Estrella; LN, La Negrita; NA, no agriculture; NT, no till farming; R, rhizospheric soil; RP, rolling pampas. Click here for file

## References

[B1] Satorre EH Slafer GA In Wheat Wheat production systems of the Pampas 1999 New York: The Haworth Press, Inc: Ecology and Physiology of Yield Determination 333 348

[B2] FAOSTAT 2013 [http://faostat.fao.org/] data sourced January 2013

[B3] Kovalevski E García F Norte de argentina bajo siembra directa 2007 1 159 172

[B4] Phillips RE Thomas GW Blevins RL Frye WW Phillips SH No-tillage agriculture Science 1980 1 1108 1113 10.1126/science.208.4448.1108 17783055

[B5] Gebhardt MR Daniel TC Schweizer EE Allmaras RR Conservation tillage Science 1985 1 625 630 10.1126/science.230.4726.625 17797277

[B6] Montgomery DR Soil erosion and agricultural sustainability Proc Nat Acad Sci USA 2007 1 13268 13272 10.1073/pnas.0611508104 17686990 PMC1948917

[B7] Alvarez R Santanatoglia OJ García R Soil respiration and carbon inputs from crops in a wheat-soyabean rotation under different tillage systems Soil Use Manage 1995 1 45 50 10.1111/j.1475-2743.1995.tb00495.x

[B8] Gomez E Bisaro V Conti M Potential C-source utilization patterns of bacterial communities as influenced by clearing and land use in a vertic soil of Argentina Applied Soil Ecol 2000 1 273 281 10.1016/S0929-1393(00)00078-0

[B9] Aon M Cabello M Sarena D Colaneri A Franco M Burgos J Cortassa S I. Spatio-temporal patterns of soil microbial and enzymatic activities in an agricultural soil Applied Soil Ecol 2001 1 239 254 10.1016/S0929-1393(01)00153-6

[B10] Nievas F Bogino P Nocelli N Giordano W Genotypic analysis of isolated peanut-nodulating rhizobial strains reveals differences among populations obtained from soils with different cropping histories Applied Soil Ecol 2012 1 74 82

[B11] Agaras B Wall LG Valverde C Specific enumeration and analysis of the community structure of culturable pseudomonads in agricultural soils under no-till management in Argentina Applied Soil Ecol 2012 1 305 319

[B12] Zabaloy MC Garland JL Gómez MA An integrated approach to evaluate the impacts of the herbicides glyphosate, 2,4-D and metsulfuron-methyl on soil microbial communities in the Pampas region, Argentina Applied Soil Ecol 2008 1 1 12 10.1016/j.apsoil.2008.02.004

[B13] Zabaloy MC Gómez E Garland JL Gómez MA Assessment of microbial community function and structure in soil microcosms exposed to glyphosate Applied Soil Ecol 2012 1 333 339

[B14] Smalla K Oros-Sichler M Milling A Heuer H Baumgarte S Becker R Neuber G Kropf S Ulrich A Tebbe CC Bacterial diversity of soils assessed by DGGE, T-RFLP and SSCP fingerprints of PCR-amplified 16S rRNA gene fragments: do the different methods provide similar results? J Microbiol Methods 2007 1 470 479 10.1016/j.mimet.2007.02.014 17407797

[B15] Muyzer G Smalla K Application of denaturing gradient gel electrophoresis (DGGE) and temperature gradient gel electrophoresis (TGGE) in microbial ecology Antonie van Leeuwenhoek 1998 1 127 141 10.1023/A:1000669317571 9602286

[B16] Sogin ML Morrison HG Huber JA Mark Welch D Huse SM Neal PR Arrieta JM Herndl GJ Microbial diversity in the deep sea and the underexplored ‘rare biosphere’ Proc Nat Acad Sci USA 2006 1 12115 12120 10.1073/pnas.0605127103 16880384 PMC1524930

[B17] Vogel TM Simonet P Jansson JK Hirsch PR Tiedje JM Van Elsas JD Bailey MJ Nalin R Philippot L TerraGenome: a consortium for the sequencing of a soil metagenome Nat Rev Microbiol 2009 1 252 252 10.1038/nrmicro2119

[B18] Yin C Jones KL Peterson DE Garrett KA Hulbert SH Paulitz TC Members of soil bacterial communities sensitive to tillage and crop rotation Soil Biol Biochem 2010 1 2111 2118 10.1016/j.soilbio.2010.08.006

[B19] Roesch LFW Fulthorpe RR Riva A Casella G Hadwin AKM Kent AD Daroub SH Camargo FAO Farmerie WG Triplett EW Pyrosequencing enumerates and contrasts soil microbial diversity ISME J 2007 1 283 290 18043639 10.1038/ismej.2007.53 PMC2970868

[B20] Delmont TO Prestat E Keegan KP Faubladier M Clark IM Pelletier E Hirsch PR Meyer F Gilbert JA Le Paslier D Simonet P Vogel TM Structure, fluctuation and magnitude of a natural grassland soil metagenome ISME J 2012 1 1677 1687 10.1038/ismej.2011.197 22297556 PMC3498926

[B21] Caporaso JG Kuczynski J Stombaugh J Bittinger K Bushman FD Costello EK Fierer N Peña AG Goodrich JK Gordon JI Huttley GA Kelley ST Knights D Koenig JE Ley RE Lozupone CA McDonald D Muegge BD Pirrung M Reeder J Sevinsky JR Turnbaugh PJ Walters WA Widmann J Yatsunenko T Zaneveld J Knight R QIIME allows analysis of high-throughput community sequencing data Nature Methods 2010 1 335 336 10.1038/nmeth.f.303 20383131 PMC3156573

[B22] Huson DH Auch AF Qi J Schuster SC MEGAN analysis of metagenomic data Genome Res 2007 1 377 386 10.1101/gr.5969107 17255551 PMC1800929

[B23] Arndt D Xia J Liu Y Zhou Y Guo AC Cruz JA Sinelnikov I Budwill K Nesbø CL Wishart DS METAGENassist: a comprehensive web server for comparative metagenomics Nucleic Acids Res 2012 1 W88 W95 10.1093/nar/gks497 22645318 PMC3394294

[B24] Rho M Tang H Ye Y FragGeneScan: predicting genes in short and error-prone reads Nucleic Acids Res 2010 1 e191 10.1093/nar/gkq747 20805240 PMC2978382

[B25] Soil Genetic Network [http://soilgene.net]

