# Supplementary material for: The PAMPA datasets: a metagenomic survey of microbial communities in Argentinean pampean soils
Source: Microbiome. 2013 Jul 29;1:21. doi: 10.1186/2049-2618-1-21 (PMC3971610; doi:10.1186/2049-2618-1-21)
Supplement: Additional file 1 — Supplemental methods. Detailed description of all materials and methods used to generate and analyze the PAMPA datasets. [file 2049-2618-1-21-S1.doc]

**Supplemental Methods Document**

**Experimental design**

Soil samples were obtained from five different locations of Argentinean Pampas, located at the central region of the country (Figure 1). Three geographic regions (Rolling Pampas, Balcarce and Anguil) were selected according to previously defined isohyets: wet weather (1,000 – 1,200 annual mm), semi-wet (800–1,000 annual mm) and semi-arid (600–800 annual mm) (Figure 1).

Sampling in Rolling Pampa was performed in three different production fields, La Negrita, LN (−34º 34´ 46,2", -59º 55´ 8,4"), La Estrella, LE (−34º 43', -60º 4´ 45") and Criadero Klein, CK (−35º 7´17,4 ", -60º 15´ 42,6") in June 2010. Rolling Pampas soils are classified as Typical Argiudolls [1]. Mean annual precipitation and temperature are 1002 mm and 16.8 °C respectively. Bulk soils from agricultural and no-agricultural plots were sampled on each site. Agricultural soils were managed under No-till farming the last 16 years and farmed for the last 30 years using a wheat-soybean-corn rotation. The last crop rotation before sampling was wheat-soybean and samples were taken a month after soybean harvest. Non-agricultural samples were obtained from grassland areas nearby the farmhouses covered with herbaceous (non-woody) plants common in the region such as *Cirsium* sp, *Trifolium* sp, *Micropsis* sp, *Festuca* sp, *Dichondra* sp, *Cyperus* sp and *Taraxacum officinale* among others grasses. In addition, La Estrella agricultural and no-agricultural plots were also sampled in a time point series on June 2009, a month after soybean harvest, on September 2009, 45 days after wheat sowing, on December 2009, two weeks after wheat harvest, on March 2010 before soybean flowering and September 2010, after soybean harvest again. See Additional File 1 for detailed description of number of replicates (i.e., independent soil samples) per plot.

Balcarce sampling location was a 34-year crop rotation experiment located in the Southern Pampas (−37º45´09´´, -58º17´47´´). Soils in Balcarce are classified as a complex of Typical Argiudolls and Petrocalcic Paleudols [2] and mean annual precipitation and temperatures are 875 mm and 13.8 °C respectively. The experiment was carried out as a randomized complete block design with a split-plot treatment arrangement, three replicates, and 10 by 35 m experimental units. Treatments assigned to main plots were referred to as no-till or conventional tillage (under mouldboard plough). Rotations have included pastures and grain crops during the last 16 years. Last rotation before sampling was corn-soybean. Bulk soil samples were collected on August 2010 from all treatments and replicates, a month after soybean harvest. Soil samples were also taken in three different areas nearby the farmhouses where no land use has been recorded in the last 30 years except from grass mowing. This area was covered with grass and other herbaceous (non-woody) plants common in the region. See Additional File 1 for detailed description of number of replicates (i.e., independent soil samples) per plot.

Anguil sampling site (−36° 35' 31.56", -63° 57' 31.05") is an experimental station located in an area with entic haplustolls soil [2]. Mean annual precipitation and temperature are 714.6 mm and 15.7 °C respectively. Sampling was performed on September 2011 a month after wheat sowing. Three different plots that were managed under conventional tillage (CT) for the last 60 years (plot 3A with a surface of 6 ha, 9A and 9B with 66ha each) and one plot that was under no-till farming (NT) for the last 4 years before sampling and under agricultural management for more that 15 years (Plot 6D with a surface of 12ha) were sampled. Two plots with a surface of 2ha each and covered by weeping lovegrass (*Eragrostis curvula*) for the last 60 years and one plot covered with herbaceous non-woody plants typical from the area were sampled as No Agriculture (NA) controls. Given that there was only one NT plot (with a surface of 12ha) at the experimental station with similar physicochemical conditions to the CT sampled plots, sample collection was performed on three different mutually distant areas (over 150 m) within the plot. The three areas were considered as independent plots. Bulk soil was sampled on each NT, CT and NA plots. Additionally, rhizospheric soil was obtained from five wheat plants collected on each sampled spots at NT and CT plots.

Metadata associated to all samples included in these datasets are provided in Additional File 2.

**Sampling protocol and storage**

Bulk soils from all sampled plots mentioned above were collected with an auger from the upper 20 cm soil layer. Each soil replicate (see Additional File 1 for a detail description of soil replicates) was obtained by pooling and mixing soils from 20 randomly selected spots in an area of ~500 m2 within the plot. Composite samples were immediately sent to the lab where they were air dried and sieved through 1 mm mesh to thoroughly homogenize, break aggregates and remove roots and plant detritus.

Plants and surrounding soil were taken with a shovel and sent to the lab for rizhospheric soil collection. Soil loosely attached to roots was disaggregated with gloved hands. Then, rhizospheric soil tightly attached to roots was obtained by brush scrapping.

Soil samples were stored at -80C immediately after homogenization until nucleic acid preparation.

**Nucleic acid isolation**

Total DNA was obtained from all bulk and rhizospheric samples using PowerSoil DNA isolation kit (MO BIO Laboratories, Inc, CA, USA) following manufacturer´s instructions. Samples used for amplicon and shotgun libraries were obtained from 10g of soil. Samples used exclusively for PCR amplification were obtained from 120 mg of soil. Total RNA was obtained from NT and CT Anguil-rhizospheric samples from 1.7g of soil using PowerSoil Total RNA purification kit (MO BIO Laboratories, Inc, CA, USA) according to manufacturer’s manuals. Single strand cDNA (cDNAss) was prepared from an aliquot of total RNA for further PCR amplification of 16S rRNA genes using First-strand cDNA synthesis system kit (Roche, Cat. 11483188001). Reagents mix and thermocycler program was done following manufacturers manuals. Purification of prokaryotic mRNA from Total RNA was performed using MicrobeExpress kit (Life Tech. Cat. AM1905) and double-stranded cDNA was prepared from mRNA using cDNA synthesis system kit (Roche Cat. 11117831001).

**Amplification**

The V4 hyper variable region of the 16s rDNA/rRNA gene was amplified using 563F-802R universal primers containing the Roche 454 sequencing A and B adaptors (underlined) and a nucleotide “multiple identifier” (MID) to sort samples (bold): 563F: **5’**‐CGTATCGCCTCCCTCGCGCCATCAG**ACGAGTGCGT**AYTGGGYDTAAAGNG ‐3’ (where **ACGAGTGCGT** is an example, different MIDs for each sample were used. See Additional File 2 for details) and 802R (5’‐CTATGCGCCTTGCCAGCCCGCTCAGTACCRGGGTHTCTAATCC, 5’‐CTATGCGCCTTGCCAGCCCGCTCAGTACCAGAGTATCTAATTC, 5’‐CTATGCGCCTTGCCAGCCCGCTCAGCTACDSRGGTMTCTAATC, 5’‐CTATGCGCCTTGCCAGCCCGCTCAGTACNVGGGTATCTAATCC) from RDP’s Pyrosequencing Pipeline: http://pyro.cme.msu.edu/pyro/help. For each sample, from two to five independent PCRs were performed and then pooled to reduce PCR bias. The PCR mix (final volume 25 ul) contained 2.5 ul FastStart High Fidelity 10X Reaction Buffer (Roche Applied Science*,* Mannheim*,* Germany), 20 ng of template DNA, 0.4 uM each primer, 1.25 U FastStart High Fidelity Enzyme Blend (Roche Applied Science), and 0.2 mM DNTPs. The PCR conditions were 95 °C for 5 minutes for initial denaturalization, followed by 95 °C for 45 seconds, 57 °C for 45 seconds, 72 °C for 60 seconds in 30 cycles, and a final elongation step at 72°C for 4 minutes. Two negative control reactions containing all components were performed without template.

Samples marked with asterisk in Additional File 2 were amplified in two rounds of PCR. First round was performed with 563F-802R 16S rRNA primers fused at 5´end to a TAG sequence (TAG-fw: CACGACGTTGTAAAACGAC in primer forward and TAG-Rv: CAGGAAACAGCTATGACC in primer reverse). PCR mix and thermocycler program was the same as described above. A second round of PCR was performed to add Roche 454 adaptors and a specific MID to each sample. Forward primers used in second round were (from 5´to 3´): Roche 454 A adaptor, MID, TAG-fw and Reverse primer was: Roche 454 B adaptor, TAG-rv. Two independent PCRs per sample were performed for first PCR round and products were pooled and used as template for two independent PCR reactions in the second round of PCR. Anguil Bulk samples marked with a dagger symbol in additional file 2 were amplified in 25 instead of 30 PCR cycles.

**Library preparation and sequencing**

PCR products were purified using Ampure DNA capture beads (Agencourt, Cat N: A63880) to eliminate primers and impurities and quantified using Quant-iT PicoGreen dsDNA Kit (Invitrogen Molecular Probes Inc, Oregon, USA). We generated one library pool for 68 samples from La Estrella, La Negrita, Criadero Klein and Balcarce by mixing purified PCR products in equal amounts and one other pool for La Estrella, La Negrita, Criadero Klein samples used for high-coverage sequencing (samples not marked with asterisk in Additional file 2). In addition, we generated one library pool for Anguil samples and one pool for La Estrella time point series.

One DNA sample from each site and condition was used to prepare Metagenomic Shotgun Libraries (See Additional File 2 for details). Rapid libraries were constructed from 500ng of DNA following Roche 454-FLX manual’s instructions. Libraries from Anguil rhizospheric cDNA samples were generated with no fragmentation step, as double strand cDNA fragment distribution was appropriate for Roche Rapid Library protocol.

Amplicon, metagenome and metatranscriptome shotgun libraries were amplified through emulsion-based clonal amplification (emPCR), and sequenced on a Genome Sequencer FLX (Roche Applied Science) using Titanium Chemistry according to the manufacturer’s instructions at INDEAR (Argentina) Genome sequencing facility. Roche manuals used were the following: Rapid Library Preparation Method Manual (Oct. 2010) to prepare shotgun libraries, Amplicon Library Preparation Method Manual (Oct. 2009) to prepare amplicon libraries, emPCR Method Manual – Lib-L LV (Jan. 2010) for bead capture and emulsion amplification of shotgun libraries, emPCR Method Manual – Lib-A LV (Jan. 2010) for bead capture and emulsion amplification of amplicon libraries and Sequencing Method Manual (Nov. 2010) to sequence all samples.

**Read quality assessment**

Raw data was processed for image processing, signal correction, basecalling and Run metrics generation by gsRunprocessor software provided by Roche using default parameters. Amplicon or shotgun processing was used according to the type of library sequenced. SFF files generated after processing were directly uploaded to SRA sequence archive. Sequences were checked for quality prior to data analysis presented in this paper using Prinseq (http://prinseq.sourceforge.net/) software. Amplicon sequences were demultiplexed and trimmed by quality using split_libraries.py script from QIIME software package [3]. Sequences longer that 500bp and shorter that 200bp, with ambiguous base pairs, mean quality score below 27 and homopolymers longer that 6 bp were excluded from the analysis.

**Data analysis**

Amplicon sequences were analyzed using QIIME package [3]. Sequences were clustered into Operational Taxonomic Units (OTUs) using the pick_otus.py script and Uclust [4] method at 90% similarity. Some of the Anguil Bulk samples (9 samples, see Additional file 2) were excluded from the analysis as we observed that differences in PCR cycles (25 vs. 30) introduced more variability than any other of the variables studied. We also excluded from the analysis 14 samples from La Negrita, La Estrella and Criadero Klein that were sequenced at a higher depth (18.000 to 142.000 reads per sample vs 560 to 7000 reads in the other samples, see Additional file 2). We observed that sequencing depth introduced a bias that strongly affected the subsequent analysis. OTU table was imported to METAGENassist online tool [5] and analysis was performed using tools provided. The first step was to filter out OTUs with more than 92 percent zeroes across samples and OTUs that were near-constant throughout the tables using Interquantile Range option (IQR). The interquartile range is first calculated individually for each OTU across all samples. Then the taxa are ranked from highest to lowest IQR values, and OTUs with the lowest IQR values (i.e. lowest variability across samples) are removed. The second step was normalization using total sum option for samples (i.e.: ensure that same number of sequences per sample are used) and range scaling option for OTUs (mean-centered and divided by the range of each OTU). Range scaling is used to make OTUs comparable to each other taking into account variability across samples rather than the absolute abundance. Heatmap analysis was performed using Pearson distance and Ward clustering algorithm. Normalized OTU tables were imported from METAGENassist to R software. PCA and ANOSIM analysis were performed based on Euclidean distances. Bioenv [6] and Mantel tests were calculated to find community drivers. Vegan and BiodiversityR packages were used for numerical and statistical analyses.

Gene prediction from Metagenome Shotgun datasets was done using FragGeneScan 7]. Predicted peptides were annotated using BlastP against NCBI nr database. Hits with evalues higher than 1E-05 were filtered out and results were imported into MEGAN [8]. Annotation by SEED categories in MEGAN was used for further analysis. Seed-category tables were imported into METAGENassist and analysis was performed as follows: 1) Filtering out SEED categories with more than 80 percent zeroes and variables that were near-constant throughout the tables by Interquantile Range, 2) Normalization by sum for samples and by range scaling for SEED categories 3) Heatmap analysis using Pearson distance and Ward clustering algorithm ,4) PCA and ANOSIM analysis in R software using METAGENassist normalized tables. 5) Bioenv and Mantel test to elucidate the contribution of soil properties on functional variability. An average of 76% rRNA was obtained from metatranscriptome shotgun libraries. Prediction of rRNA was performed on CAMERA Portal (https://portal.camera.calit2.net) using Blast and Hmmer methods. Due to the low number of reads recovered after rRNA trimming, metatranscriptomic samples were excluded from the analysis described above as only few and most abundant SEED categories could be detected at this sequence depth.

**References**

1. Lavado R**: La Región Pampeana: historia, características y uso de sus suelos**. *Materia Orgánica. Valor agronómico y dinámica en suelos pampeanos.* *(Editorial Facultad de Ingenieria, Buenos Aires)* 2006 pp. 1–11

2. . INTA: **Carta de Suelos de la República Argentina.** *Instituto Nacional de Tecnología Agropecuaria (Secretaría de Agricultura, Ganadería y Pesca, Buenos Aires)* 1979 pp. 3757–3831.

3. Caporaso JG, Kuczynski J, Stombaugh J, Bittinger K, Bushman FD, Costello EK, Fierer N, Peña AG, Goodrich JK, Gordon JI, Huttley GA, Kelley ST, Knights D, Koenig JE, Ley RE, Lozupone CA, Mcdonald D, Muegge BD, Pirrung M, Reeder J, Sevinsky JR, Turnbaugh PJ, Walters WA, Widmann J, Yatsunenko T, Zaneveld J, Knight R: **QIIME allows analysis of high- throughput community sequencing data**. *Nature Methods* 2010, **7**:335–336.

4. Edgar RC: **Search and clustering orders of magnitude faster than BLAST.** *Bioinformatics (Oxford, England)* 2010, **26**:2460–1.

5. Arndt D, Xia J, Liu Y, Zhou Y, Guo AC, Cruz J a, Sinelnikov I, Budwill K, Nesbø CL, Wishart DS: **METAGENassist: a comprehensive web server for comparative metagenomics.** *Nucleic acids research* 2012, **40**:W88–95.

6.Clarke, K. R & Ainsworth, M. **A method of linking multivariate community structure to environmental variables.** Marine Ecology Progress Series, 1993, 92: 205–219.

7. Rho M, Tang H, Ye Y: **FragGeneScan: predicting genes in short and error-prone reads.** *Nucleic acids research* 2010, **38**:e191.

8. Huson DH, Auch AF, Qi J, Schuster SC: **MEGAN analysis of metagenomic data.** *Genome research* 2007, **17**:377–86.
