# Supplementary material for: The PAMPA datasets: a metagenomic survey of microbial communities in Argentinean pampean soils
Source: Microbiome. 2013 Jul 29;1:21. doi: 10.1186/2049-2618-1-21 (PMC3971610; doi:10.1186/2049-2618-1-21)
Supplement: Additional file 3: Figure S1 — Heatmap and beta-diversity analysis for amplicon and metagenome shotgun libraries in PAMPA datasets. (A) A total of 103 soil samples were analyzed by 16S rDNA/rRNA V4 amplicon sequencing. Sequences were clustered in OTUs at 90% similarity. Low abundance and infrequent OTUs were excluded from the analysis (see Additional file 1 for a detailed description of the filtering procedures). Datasets were normalized and compared using the Pearson distance and Ward clustering algorithm. The scale bar at the top is expressed according to the range of values after normalization. Metadata for each sample are indicated by color bars at the right and references are indicated at the top. (B) A total of 30 soil samples were analyzed by metagenomic shotgun sequencing. Predicted peptides were annotated by BlastP against the NCBI database and the results assigned to SEED categories. Low abundance and infrequent SEED categories were excluded from the analysis (see Additional file 1). Datasets were normalized and compared using the Pearson distance and Ward clustering algorithm. Metadata are indicated with same references as in A. An, Anguil; B, bulk soil; Ba, Balcarce; CK, Criadero Klein; CT, conventional tillage; LE, La Estrella; LN, La Negrita; NA, no agriculture; NT, no till farming; R, rhizospheric soil; RP, rolling pampas. [file 2049-2618-1-21-S3.pdf]

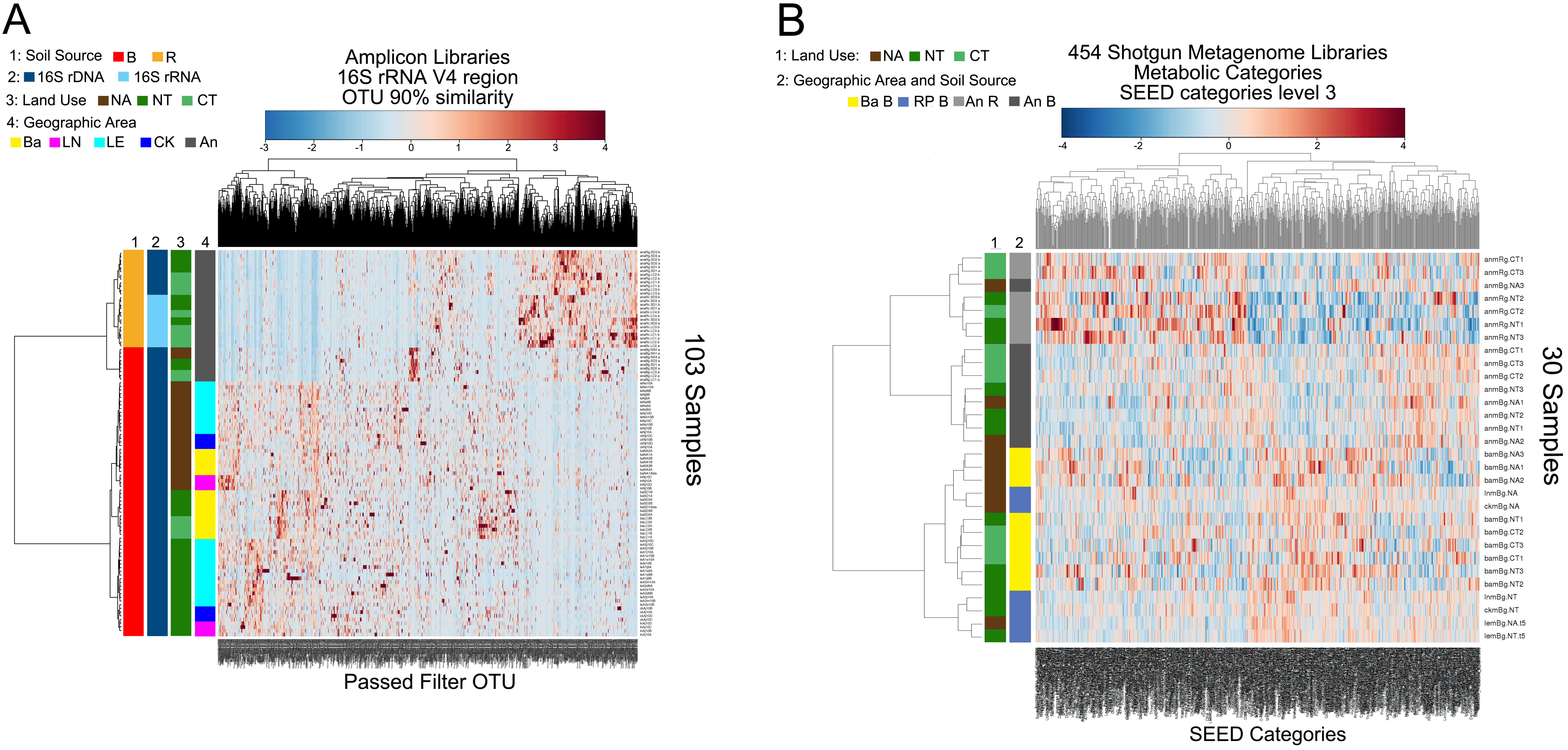
A
1: Soil Source B R
2: 16S rDNA 16S rRNA
3: Land Use NA NT CT
4: Geographic Area
Ba LN LE CK An
Amplicon Libraries
16S rRNA V4 region
OTU 90% similarity
-3 -2 -1 0 1 2 3 4
1 2 3 4
103 Samples
Passed Filter OTU
B
1: Land Use: NA NT CT
2: Geographic Area and Soil Source
Ba B RP B An R An B
454 Shotgun Metagenome Libraries
Metabolic Categories
SEED categories level 3
-4 -2 0 2 4
1 2
anmRg.CT1
anmRg.CT3
anmBg.NA3
anmRg.NT2
anmRg.CT2
anmRg.NT1
anmRg.NT3
anmBg.CT1
anmBg.CT3
anmBg.CT2
anmBg.NT3
anmBg.NA1
anmBg.NT2
anmBg.NT1
anmBg.NA2
bamBg.NA3
bamBg.NA1
bamBg.NA2
lnmBg.NA
ckmBg.NA
bamBg.NT1
bamBg.CT2
bamBg.CT3
bamBg.CT1
bamBg.NT3
bamBg.NT2
lnmBg.NT
ckmBg.NT
lemBg.NA.t5
lemBg.NT.t5
30 Samples
SEED Categories
